# Systemic pain relief after omalizumab injection in patient with hypermobile Ehlers–Danlos syndrome: A case report

**DOI:** 10.1002/ccr3.8431

**Published:** 2024-01-12

**Authors:** Sarahrose Jonik, Andrew Joseph Rothka, Neyha Cherin

**Affiliations:** ^1^ Department of Physical Medicine and Rehabilitation Penn State College of Medicine Hummelstown Pennsylvania USA

**Keywords:** chronic pain, hypermobile Ehlers–Danlos syndrome, omalizumab, physical medicine and rehabilitation, quality of life

## Abstract

Omalizumab may be a beneficial adjunct treatment option for hEDS patients require to improve pain control, ability to perform ADLs and functionality and social engagement, and most importantly, quality of life.

## Graphical abstract

Symptomatology pre and post Omalizumab treatment.

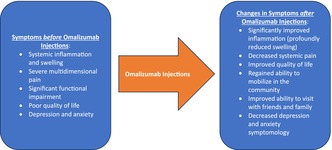

Treating pain in patients with hypermobile Ehlers–Danlos syndrome (hEDS) is complex and must be addressed in a multidisciplinary fashion. Patients tend to experience diffuse, chronic, debilitating pain. Pain is described as both nociceptive and neuropathic in nature and is frequently systemic in presentation. We present a case of a 33‐year‐old hEDS female patient with severe multi‐dimensional pain, resulting in significant functional impairment, poor quality of life based on subjective scores, and severe isolation due to her uncontrolled symptomatology. Additionally, she has a past medical history of chronic regional pain syndrome (CRPS), chronic idiopathic urticaria, genetically confirmed hereditary alpha tryptasemia, gastroparesis, small fiber neuropathy, ureteral stones, depression, and fibromyalgia. She follows with the Physical Medicine and Rehabilitation (PM&R) clinic for ongoing treatment of hEDS and comorbid conditions (Mast Cell Activation Syndrome [MCAS] and Postural Orthostatic Tachycardia Syndrome [POTS]) with the goal of improved independence, functionality, and quality of life.

Despite many treatment trials, both pharmacologic and non‐pharmacologic in nature, the patient's chronic systemic symptoms remained uncontrolled. The PM&R team was further limited on additional pharmacologic treatments due to her significant allergy history including gabapentin, zolpidem, ramelteon, duloxetine, eszopiclone, pregabalin, sulfamethoxazole‐trimethoprim, clonidine, cortisone, erythromycin, melatonin, nortriptyline, ropinirole, and trazodone. From a non‐pharmacological perspective, the patient was unable to tolerate an anti‐inflammatory diet due to comorbid gastroparesis, and failed physical and cognitive behavior therapies due to pain limitations and financial concerns. The PM&R team and patient continuously faced a vicious cycle of trialing and failing a range of treatment modalities, appropriately managing healthcare spending and facing the psychological impact of being able to neither provide or receive assistance in a desperate situation.

In January 2023, a breakthrough in this cycle was noted once the patient began receiving omalizumab injections by her Allergy and Immunology provider. Given her history of genetically confirmed hereditary alpha tryptasemia, chronic idiopathic urticaria, and poorly controlled MCAS on prior failed medical regiments, Allergy and Immunology felt starting omalizumab injections would be the appropriate next step. Omalizumab is a monoclonal antibody that binds to IgE receptors on mast cells and basophils to prevent an immune‐mediated reaction.^1^ After receiving five injections, the patient reported significant improvement in the inflammation throughout her body, with additional profound reduction in her systemic swelling. Subsequently, the patient noticed a decrease in systemic pain and significant increase in daily functioning.

The patient continues to follow with the PM&R department and has maintained an overall reduction in limiting systemic symptoms and chronic pain. This has been tracked with the visual analog scale (VAS) at each appointment. Pre‐omalizumab treatment, pain was recorded as 10/10. Post‐omalizumab treatment, pain has consistently been recorded as 6–7/10. As noted in literature, a 3‐point reduction on the VAS is a clinically significant outcome correlating to patient perception of sufficient pain control.^2^ While omalizumab has been beneficial in decreasing systemic inflammation and pain, this most importantly has led to an increase in the patient's functional mobility and independence. The patient notes improved ability to perform activities of daily living (ADLs), mobilize in the community, visit with friends and family, and a decrease in her overall depression and anxiety symptomatology.

Ultimately, the reduction in this patient's systemic inflammatory symptoms and chronic pain after initiating omalizumab therapy led the authors to question of possible links between antihistamines, omalizumab, and chronic pain relief. A literature search was conducted, and to the author's knowledge, there is no prior literature researching this connection.

Pain is a hallmark of inflammation, and there is considerable evidence that suggests that mast cells play a role in the pain signaling pathway by secreting mediators that induce peripheral sensitization. Targeting mast cell activation and production may improve analgesic therapy for systemic mast cell activation disease.^3^ Currently, omalizumab is approved by the Food and Drug Administration for use in cases of mast cell activation syndrome, urticaria, and severe allergic asthma.^4^, ^5^

In patients with hEDS, uncontrolled, diffuse, atypical pain is often the presenting symptom, multi‐dimensional in nature, and subsequently the most difficult to treat. While there are multiple factors that potentially contribute to this complaint, including nociceptive pain, neuropathic pain, impaired proprioception, and central sensitization, the mechanism behind pain in hEDS and its subtypes is poorly understood.^6^ Therefore, there are no disease‐modifying treatments available for hEDS, rather management is tailored to the individual patient's symptomatology.^7^ Current methods in practice include physiotherapy, pharmacologic pain management, and lifestyle modifications, including limited vigorous activity to decrease bone and muscle morbidity.^8^, ^9^

In addition, the role of the immune system in any of the EDS subtypes is not well known. Few case reports suggest a possible association between hEDS, allergies, and immunodeficiencies^10^; however, further research is required to better understand the correlation.^11^ In addition to the commonly observed rheumatological manifestations of hEDs, such as joint instability, arthralgias, myalgias, and arthritis, many patients with hEDS have comorbid systemic inflammatory disorders that could be explained by a chronic release of mast cell‐related immune factors.^12^ Mast cells are known to express IgE and IgG receptors, among several others, that release proinflammatory granule mediators like histamine upon binding. When unregulated, as seen in hEDS, these mediators can affect virtually any organ system and therefore present with a wide range of clinical manifestations, including IBS, urticaria, hypotension, and cardiac arrhythmias,^4^, ^13^ as in the case of our patient.

Fortunately, our patient with hEDS and MCAS was finally able to receive relief of her wide‐spread symptomatology after starting omalizumab and subsequently experienced a significant improvement in functionality, patient satisfaction, and quality of life. Though omalizumab can be used to treat comorbidities of hEDS, we propose there may be use in considering further research in applying omalizumab as a potential therapy in hEDS patients with uncontrolled systemic pain. Further research can evaluate the relationship between omalizumab and pain in hEDS more closely with the potential for omalizumab to reduce pain in hEDS. If found beneficial with further research, we hope for earlier integration of omalizumab into the multi‐dimensional treatment regimen hEDS patients require to improve pain control, ability to perform ADLs and functionality and social engagement, and most importantly, quality of life.

## AUTHOR CONTRIBUTIONS

**Sarahrose Jonik:** Conceptualization; investigation; writing – original draft; writing – review and editing. **Andrew Joseph Rothka:** Conceptualization; investigation; writing – original draft; writing – review and editing. **Neyha Cherin:** Conceptualization; investigation; project administration; writing – original draft; writing – review and editing.

## FUNDING INFORMATION

No source of funding.

## CONFLICT OF INTEREST STATEMENT

No conflicts of interest.

## CONSENT

Written informed consent was obtained from the patient to publish this report in accordance with the journal's patient consent policy.

## DECLARATION

All protected health information was withheld from this piece to ensure patient anonymity.

## Data Availability

Data sharing is not applicable to this article as no datasets were generated or analyzed during the current study.

## References

[1] Kumar C , Zito PM . Omalizumab. In: Stat Pearls [Internet]. Treasure Island (FL). January 2023. https://www.ncbi.nlm.nih.gov/books/NBK545183/

[2] Lee JS , Hobden E , Stiell IG , Wells GA . Clinically important change in the visual analog scale after adequate pain control. Acad Emerg Med. 2003;10(10):1128‐1130. 14525749 10.1111/j.1553-2712.2003.tb00586.x

[3] Mai L , Liu Q , Huang F , He H , Fan W . Involvement of mast cells in the pathophysiology of pain. Front Cell Neurosci. 2021;10(15):665066. 10.3389/fncel.2021.665066 PMC8222580 34177465

[4] Monaco A , Choi D , Uzun S , Maitland A , Riley B . Association of mast‐cell‐related conditions with hypermobile syndromes: a review of the literature. Immunol Res. 2022;70(4):419‐431. 35449490 10.1007/s12026-022-09280-1 PMC9022617

[5] El‐Qutob D . Off‐label uses of Omalizumab. Clin Rev Allergy Immunol. 2016;50(1):84‐96. 26048266 10.1007/s12016-015-8490-y

[6] Syx D , De Wandele I , Rombaut L , Malfait F . Hypermobility, the Ehlers‐Danlos syndromes and chronic pain. Clin Exp Rheumatol. 2017;35(5):116‐122. 28967365

[7] The Ehlers‐Danlos Society. 2023. https://www.ehlers‐danlos.com/

[8] Chopra P , Tinkle B , Hamonet C , et al. Pain management in the Ehlers‐Danlos syndromes. Am J Med Genet C Semin Med Genet. 2017;175(1):212‐219. 28186390 10.1002/ajmg.c.31554

[9] Miklovic T , Sieg VC . Ehlers‐Danlos syndrome. StatPearls. StatPearls Publishing; 2023. 31747221

[10] Wang E , Ganti T , Vaou E , Hohler A . The relationship between mast cell activation syndrome, postural tachycardia syndrome, and Ehlers‐Danlos syndrome. Allergy Asthma Proc. 2021;42(3):243‐246. 33980338 10.2500/aap.2021.42.210022

[11] Islam M , Chang C , Gershwin ME . Ehlers‐Danlos syndrome: immunologic contrasts and connective tissue comparisons. J Transl Autoimmun. 2020;4:100077. 33437956 10.1016/j.jtauto.2020.100077 PMC7786113

[12] Castori M , Tinkle B , Levy H , Grahame R , Malfait F , Hakim A . A framework for the classification of joint hypermobility and related conditions. Am J Med Genet C Semin Med Genet. 2017;175(1):148‐157. 28145606 10.1002/ajmg.c.31539

[13] Hamilton MJ . Nonclonal mast cell activation syndrome: a growing body of evidence. Immunol Allergy Clin North Am. 2018;38(3):469‐481. 30007464 10.1016/j.iac.2018.04.002 PMC6049091

